# Web-Based Mindfulness Intervention for Reducing Perceived Stress in Health Care Professionals: Secondary Analysis of Moderators in a Stepped-Wedge Cluster Randomized Trial

**DOI:** 10.2196/98624

**Published:** 2026-08-14

**Authors:** Gloria Guerrero-Pertiñez, Vera Carbonell Aranda, Jonathan Joseph Dawood-Histrova, Gloria Pérez-Guerrero, Adrián Pérez-Aranda, Selene Fernández-Martínez, Alicia Monreal-Bartolomé, Alberto Barceló-Soler, Javier García-Campayo, Yolanda López del Hoyo, Jesús Herrera-Imbroda, Jessica Marian Goodman-Casanova, Jose Guzman-Parra

**Affiliations:** 1 Grupo C-03: Investigación Básica, Clínica y Epidemiológica en Salud Mental, IBIMA Plataforma BIONAND, Instituto de Investigación Biomédica de Málaga, Plaza del Hospital Civil s/n, Málaga, Andalucía, Spain, 34 655256404; 2 Hospital Universitario Virgen de la Victoria, Málaga, Andalucía, Spain; 3 Grupo C-06: Neuropsicofarmacología, IBIMA Plataforma BIONAND, Instituto de Investigación Biomédica de Málaga, Málaga, Andalucía, Spain; 4 Unidad de Gestión Clínica en Salud Mental, Hospital Regional Universitario de Málaga, Málaga, Andalucía, Spain; 5 Department of Clinical and Health Psychology, Universitat Autònoma de Barcelona, Barcelona, Catalonia, Spain; 6 Centro de Investigación Biomédica en Red de Epidemiología y Salud Pública, Madrid, Madrid, Spain; 7 Faculty of Medicine, Universidad de Zaragoza, Zaragoza, Aragón, Spain; 8 Department of Psychology and Sociology, Universidad de Zaragoza, Zaragoza, Aragon, Spain; 9 Instituto de Investigación Sanitaria Aragón, Zaragoza, Aragon, Spain; 10 Research Network on Chronicity, Primary Care and Health Promotion (RICAPPS), Zaragoza, Aragón, Spain

**Keywords:** perceived stress, health care professionals, digital intervention, mindfulness, depression, resilience, mental health

## Abstract

**Background:**

Health care professionals are exposed to high levels of occupational stress, emotional exhaustion, and anxiety-depressive symptoms. Digital interventions using mindfulness- and acceptance-based approaches have shown promising results in improving psychological well-being in this population. The MINDxYOU program is a self-guided digital intervention designed to reduce perceived stress and enhance emotional regulation, resilience, and psychological flexibility. Although its effectiveness has been demonstrated, treatment response may vary substantially between individuals.

**Objective:**

The aims of this study are to explore the sociodemographic, occupational, and psychological moderators of the effectiveness of the digital MINDxYOU program on perceived stress among health care professionals and to identify differential response profiles.

**Methods:**

This study is a secondary analysis of a stepped-wedge cluster randomized trial including 347 health care professionals from 6 clusters allocated to 3 intervention start sequences. The intervention (MINDxYOU) is a self-guided, web-based program designed to be completed over 8 weeks, grounded in third-wave psychological approaches and incorporating mindfulness-, compassion-, and acceptance-based components. Weekly support was provided via WhatsApp Messenger (Meta Platforms Inc), phone calls, or email to promote adherence. Assessments were conducted every 8 weeks across 5 measurement waves. Linear mixed-effects models were used to evaluate moderation effects by testing condition × moderator interactions, accounting for repeated measures within participants and clustering effects.

**Results:**

Moderation analyses showed greater reductions in perceived stress among participants with higher baseline depressive symptom scores (9-item Patient Health Questionnaire [PHQ-9]; B=−0.22, SE 0.08; *P*=.004) and those with higher scores in the observing facet of mindfulness (15-item Five Facet Mindfulness Questionnaire [FFMQ-15]; B=−0.86, SE 0.41; *P*=.04). In addition, nursing staff exhibited greater reductions in perceived stress compared with physicians (B=1.92, SE 0.81; *P*=.02) and other health care professionals (B=1.80, SE 0.90; *P*=.04).

**Conclusions:**

The results suggest that baseline psychological and occupational characteristics may contribute to differences in the effectiveness of the MINDxYOU program in reducing perceived stress. These findings provide preliminary evidence regarding potential response profiles in digital mental health interventions and support the development of more personalized and targeted approaches to stress management in health care settings.

## Introduction

The increasing levels of occupational stress among health care professionals represent a growing public health concern, with implications at both the individual and organizational levels. Prolonged exposure to high emotional demands, work overload, extended shifts, and ethical conflicts generates emotional exhaustion, compassion fatigue, and a high risk of burnout [1,2]. This situation has worsened in recent years due to the COVID-19 pandemic, which intensified health care pressure and increased levels of anxiety, depression, and psychological distress among health care workers [3,4].

The impact of chronic stress in this population affects not only professionals’ well-being but also quality of care, patient safety, and the sustainability of health care systems. Recent studies have shown that workers experiencing high levels of burnout are more likely to commit medical errors, show reduced empathy, and report greater intentions to leave the profession [5]. In this context, promoting the mental health and psychological resilience of health care professionals has become a strategic priority at both the institutional and scientific levels.

Stress-reduction programs delivered face-to-face have demonstrated effectiveness; however, their implementation is often limited by time constraints, rotating shifts, and the emotional demands inherent to clinical practice. In this context, digital interventions based on third-wave cognitive behavioral therapies, such as acceptance and commitment therapy, mindfulness-based interventions, and compassion-focused approaches, have demonstrated effectiveness in reducing psychological distress and improving emotional coping across different occupational and clinical settings [6,7]. These digital interventions offer several advantages, including greater accessibility, lower costs, and scalability, which facilitate their implementation in high-demand health care environments [8]. Recent evidence has further highlighted the potential of digital mental health interventions to support workforce well-being and address unmet mental health needs in occupational settings [9-11]. These findings are particularly relevant in health care settings, where digital interventions have demonstrated promising effects on stress reduction, burnout prevention, and psychological well-being among health care professionals [9,10,12-14].

Within this framework, MINDxYOU was developed as a self-guided digital program designed to promote emotional self-regulation, acceptance, and resilience among health care professionals. Results from a stepped-wedge cluster randomized trial demonstrated that MINDxYOU significantly reduced levels of stress, anxiety, and depression in this population [12]. A subsequent mediation analysis revealed that improvements in resilience constituted a significant psychological mechanism underlying the observed intervention effects [15].

However, even when interventions show overall effectiveness, treatment response may vary substantially across individuals, with previous studies also showing substantial heterogeneity in the outcomes associated with this type of intervention [16]. Increasing attention has been devoted to identifying the moderators of treatment effects, defined as personal or contextual characteristics that determine for whom and under which conditions an intervention is the most effective [9,17]. This approach has gained increasing relevance in digital mental health research, where recent evidence highlights personalization and adaptive intervention strategies as key factors for improving intervention effectiveness, engagement, and implementation outcomes [17-19].

Although the MINDxYOU program has shown beneficial effects in reducing perceived stress among health care professionals, less is known about whether these effects may differ according to participants’ baseline characteristics. Exploring potential sources of variability in response is relevant for advancing the personalization of digital mental health interventions and for improving their applicability in health care settings. Therefore, the present study aimed to examine whether individual, psychological, and occupational characteristics were associated with differential responses to the MINDxYOU program in terms of perceived stress reduction.

## Methods

### Design

This study followed a stepped-wedge cluster randomized trial design, in which 6 clusters of health care professionals were randomly assigned to 3 intervention start sequences. Each sequence began the intervention at different time points separated by 2-month intervals, ensuring that all groups eventually received the program. This design allowed comparisons between participants who had not yet accessed the intervention and those who had already completed it while maintaining temporal control of the intervention effects.

### Participants

The sample included 347 health care professionals. Inclusion criteria were (1) employment as a health care professional (eg, physician, nurse, psychologist, or nursing assistant) or enrollment in training in any health-related field, (2) age between 18 and 70 years, (3) ability to understand Spanish, (4) digital literacy and access to an internet-connected smartphone, tablet, or computer, and (5) expected continued employment in the same workplace for the following 6 months. Participants with incomplete data in the main study variables or with significant changes in their employment status during the follow-up period were excluded.

Participants were grouped into 6 clusters according to their workplace, and these clusters were randomly assigned to an intervention sequence (MINDxYOU) using a computerized simple randomization procedure. Assessments were conducted every 8 weeks across 5 time points throughout the study. At each measurement point, participants completed the same self-report questionnaires through the program’s digital platform.

### Intervention

The MINDxYOU program is a self-guided, web-based intervention consisting of 4 modules delivered over 8 weeks, integrating mindfulness-, compassion-, and acceptance-based strategies. Content was delivered through videos, audio-guided practices, and interactive exercises. Participants received weekly support via WhatsApp Messenger (Meta Platforms Inc), phone, or email to promote adherence. In the stepped-wedge design, participants in the control condition were placed on a waiting list and received the intervention at later time points. Further details on the intervention components, module contents, and delivery procedures are provided in the study protocol [20].

### Ethical Considerations

The study protocol was approved by the Research Ethics Committee of the Autonomous Community of Aragon and the Ethics and Research Committee of Northeast Malaga in July 2022 (approval code PI22/341). All participants provided informed consent prior to participation, and the study was conducted in accordance with the Declaration of Helsinki and European data protection regulations [21].

### Measures

In the MINDxYOU clinical trial, a comprehensive set of self-report instruments was administered to assess clinical and psychological variables. Detailed information regarding the structure, reliability, and validity of these measures is described in the study protocol [20].

The primary outcome was perceived stress, assessed using the 10-item Perceived Stress Scale (PSS-10; [22]; Spanish version [23]). Secondary outcome variables included (1) depressive symptoms, assessed with the 9-item Patient Health Questionnaire (PHQ-9; [24]; Spanish version [25]); (2) generalized anxiety, measured with the 7-item Generalized Anxiety Disorder ([26]; Spanish version [27]); and (3) general psychological symptoms, assessed using the 18-item Brief Symptom Inventory ([28]; Spanish version [29]).

Regarding psychological processes associated with change, mindfulness was assessed using the 15-item Five Facet Mindfulness Questionnaire (FFMQ-15; [30]; Spanish version [31]). Psychological resilience was measured using the Connor-Davidson Resilience Scale (CD-RISC; [32]; Spanish version [33]). Compassion and self-compassion were assessed using the Sussex-Oxford Compassion Scales ([34]; Spanish version [35]) and the Acceptance and Action Questionnaire-II ([36]; Spanish version [37]).

### Variable Selection

The selection of moderator variables was conducted in several stages. First, a panel of researchers defined an initial set of potential moderators based on the previous literature on stress-reduction programs for health care professionals and on the theoretical plausibility of the programs’ relationship with treatment response [38]. Subsequently, a correlation matrix was examined to assess the relationships between baseline variables and perceived stress symptoms. To avoid problems of multicollinearity and conceptual redundancy, priority was given to variables that showed moderate correlations with baseline perceived stress symptoms while representing distinct psychological or contextual constructs. Variables with correlations greater than *r*=0.65 were excluded.

Following this procedure, a final set of moderators was defined, including (1) sociodemographic variables, including age and gender; (2) work-related variables, including type of contract (temporary vs permanent), occupation (physician, nurse [nurse or nursing assistant], or other health care professional), management position (yes/no), and workplace setting (hospital, primary care center, nursing home, or other); (3) clinical variables, including depressive symptoms measured with the PHQ-9; and (4) process variables, including psychological resilience, measured with the CD-RISC, and the observing facet of mindfulness, assessed with the FFMQ-15.

Additionally, variables related to the intervention were analyzed separately in order to explore whether the level of participation and the timing of intervention initiation influenced treatment outcomes. These variables included the intervention sequence, corresponding to the different start times established by the stepped-wedge design. Adherence was also considered and was defined as completion of at least 3 (out of 4) modules among participants who initiated the program.

Thus, the main set of moderators included sociodemographic factors, occupational factors, clinical factors, and process variables, while intervention-related variables were analyzed in a separate block to provide complementary information regarding the role of program use and adherence in the effectiveness of the intervention, as well as the potential influence of delayed initiation of the intervention across sequences.

### Statistical Analysis

Data were analyzed using linear mixed-effects regression models to account for the longitudinal and clustered structure of the data. Repeated assessments were nested within participants, and participants were clustered within health care centers. The primary outcome was the PSS-10 total score. For each potential moderator, a separate mixed-effects model was fitted. Each model included the condition (intervention vs control), the moderator, and the condition × moderator interaction as fixed effects. The interaction term was used to test whether the effect of the intervention on perceived stress differed according to the baseline value or category of the moderator. All models were adjusted for baseline PSS-10 score and assessment time. Random intercepts were specified at the participant and cluster levels. Collinearity among baseline predictors was examined using a correlation matrix before fitting the models. All potential moderators were then entered into the same multivariable mixed-effects model. This model was used to examine whether each moderator was independently associated with a differential intervention response after adjusting for the remaining moderators. Intervention-related variables, including intervention sequence and adherence, were analyzed separately using mixed-effects models adjusted for the same baseline covariates. All analyses were conducted in R software (version 4.4.1; R Foundation for Statistical Computing) using the *nlme* and *lme4* packages [39]. A 2-tailed significance level of .05 was adopted for all statistical tests.

## Results

### Sample Characteristics

The final sample consisted of 347 health care professionals, including 297 (85.6%) women with a mean age of 45.01 (SD 11.2) years. Participants were classified as physicians (n=147, 42.4%), nursing staff including nurses and nursing assistants (n=93, 26.8%), and other health care professionals (n=107, 30.8%). Further details of the baseline characteristics of the overall sample and across the randomized intervention sequences are presented in Table 1.

**Table 1.** Baseline characteristics of the study sample.

Variables	Total sample (N=357)	Sequence 1 (n=138)	Sequence 2 (n=135)	Sequence 3 (n=74)
Sociodemographic characteristics				
Gender, women, n (%)	297 (85.6)	117 (84.2)	114 (84.4)	66 (89.2)
Age (years), mean (SD)	45 (11.2)	47 (11)	43.5 (10.1)	44.2 (12.8)
Work-related aspects, n (%)				
Type of contract				
Temporary contract	64 (18.4)	20 (13.9)	32 (23.2)	12 (16.0)
Permanent contract	283 (81.6)	124 (86.1)	106 (76.8)	63 (84.0)
Occupation				
Physician	147 (42.4)	63 (43.8)	50 (36.2)	34 (45.3)
Nursing staff	93 (26.8)	37 (25.7)	37 (26.8)	19 (25.3)
Other health care professional	107 (30.8)	44 (30.6)	51 (37.0)	22 (29.3)
Workplace setting				
Hospital	85 (23.8)	37 (25.7)	48 (34.8)	0 (0)
Primary care center	92 (25.8)	48 (33.3)	0 (0)	44 (58.7)
Nursing home	45 (12.6)	1 (0.7)	26 (18.8)	18 (24)
Other setting	135 (37.8)	58 (40.3)	64 (46.4)	13 (17.3)
Management role, yes	52 (15)	20 (14.5)	25 (18.5)	7 (9.5)
PSS-10^a^, mean (SD), score range 0-40	16.9 (6.3)	17.4 (6.7)	15.8 (5.9)	18 (6.3)
Secondary outcome and process variables, mean (SD)				
PHQ-9^b^, score range 0‐27	6.3 (4.4)	6.6 (4.4)	5.7 (4.3)	6.6 (4.3)
CD-RISC^c^, score range 0‐40	27.3 (6.8)^d^	27 (6.9)	28.2 (6.7)	26.3 (6.7)
Observing facet of FFMQ-15^e^, score range 1-5	2.8 (0.9)	2.9 (0.8)^d^	2.9 (0.9)	2.8 (0.9)^f^

a PSS-10: 10-item Perceived Stress Scale.

b PHQ-9: 9-item Patient Health Questionnaire.

c CD-RISC: Connor-Davidson Resilience Scale.

d One missing value.

e FFMQ-15: 15-item Five Facet Mindfulness Questionnaire.

f Two missing values.

### Moderation Analysis

**Figure 1.**
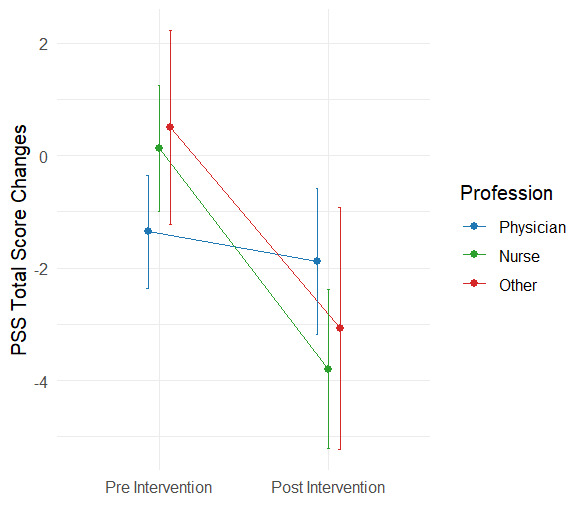
Changes in perceived stress using the 10-item Perceived Stress Scale (PSS-10) by professional category. Error bars indicate 95% CI.

Professional occupation showed a significant moderation effect. Using nursing staff as the reference category, physicians (B=1.92, SE 0.81; *P*=.02) and other health care professionals (B=1.80, SE 0.90; *P*=.04) exhibited smaller reductions in perceived stress following the intervention. Further details of the univariate moderation analyses are presented in Table 2. Figure 1 graphically illustrates the changes in perceived stress levels during the preintervention and postintervention periods across professional groups.

**Table 2.** Univariate moderation analyses of the effect of the intervention on perceived stress.

Moderator	Estimated B^a^ (SE)^b^	*t* test (*df*)	*P* value
Gender (reference: woman)	0.97 (0.82)	1.19 (497)	.24
Condition	−0.38 (1.22)	−0.31 (759)	.76
Condition × gender	−0.98 (0.95)	−1.03 (730)	.30
Age	0.02 (0.03)	0.90 (461)	.37
Condition	−1.24 (1.56)	−0.79 (775)	.43
Condition × age	−0.01 (0.03)	−0.19 (756)	.85
Employment type (temporary; reference: nontemporary)	0.07 (0.77)	0.09 (509)	.92
Condition	−1.443 (0.567)	−2.55 (814)	.01
Condition × employment	−0.30 (0.96)	−0.31 (776)	.76
Management position (reference: no)	−0.52 (0.80)	−0.65 (470)	.52
Condition	0.34 (1.22)	0.28 (767)	.78
Condition × management	−1.58 (0.95)	−1.66 (750)	.10
Workplace setting (reference: hospital)			
Primary care center	−1.22 (0.81)	−1.50 (491)	.13
Nursing home	−0.09 (0.97)	−0.09 (441)	.93
Other	−1.05 (0.75)	−1.392 (511)	.16
Condition	−1.35 (0.821)	−1.646 (766)	.10
Condition × primary care center	0.81 (0.98)	0.832 (755)	.41
Condition × nursing home	−0.76 (1.24)	−0.609 (725)	.54
Condition × other	−0.62 (0.87)	−0.717 (737)	.47
Occupation (reference: nurse)			
Physician	−0.63 (0.70)	−0.898 (481)	.37
Other	0.36 (0.77)	0.473 (483)	.64
Condition	−2.80 (0.76)	−3.674 (782)	<.001
Condition × physician	1.92 (0.81)	2.357 (741)	.02
Condition × other	1.80 (0.90)	2.014 (747)	.04
Baseline depression (PHQ-9^c^)	0.27 (0.08)	3.257 (405)	<.001
Condition	−0.082 (0.728)	−0.113 (776)	.91
Condition × baseline depression	−0.22 (0.08)	−2.872 (757)	.004
Resilience (CD-RISC^d^)	−0.142 (0.048)	−2.936 (414)	.003
Condition	−2.242 (1.41)	−1.596 (749)	.11
Condition × resilience	0.02 (0.05)	0.505 (732)	.61
Mindfulness—observing (FFMQ-15^e^)	0.170 (0.342)	0.497 (458)	.62
Condition	0.92 (1.31)	0.697 (769)	.49
Condition × mindfulness—observing	−0.86 (0.41)	−2.073 (752)	.04

a B: unstandardized regression coefficient.

b Linear mixed-effects models include assessment time and baseline perceived stress as covariates.

c PHQ-9: 9-item Patient Health Questionnaire.

d CD-RISC: Connor-Davidson Resilience Scale.

e FFMQ-15: 15-item Five Facet Mindfulness Questionnaire.

**Figure 2.**
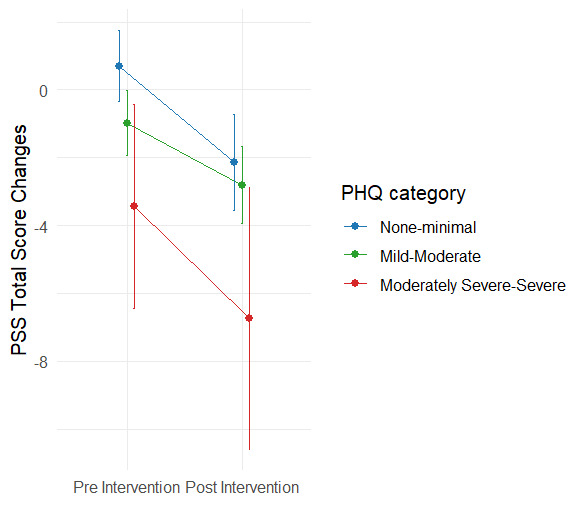
Changes in perceived stress using the 10-item Perceived Stress Scale (PSS-10) before and after the intervention by 9-item Patient Health Questionnaire (PHQ-9) depression severity category. None-minimal (0‐4 score), mild-moderate (5‐14 score), and moderately severe to severe depression (15‐27 score). Error bars indicate 95% CI.

Baseline depressive symptoms significantly moderated the intervention effect on perceived stress (B=−0.22, SE 0.08; *P*=.004). Higher baseline depression symptom scores were associated with greater reductions in perceived stress following the intervention. Further details of these analyses are presented in Table 2. Figure 2 illustrates the reductions in perceived stress during the preintervention and postintervention periods across 3 groups defined by baseline depressive symptom severity.

**Figure 3.**
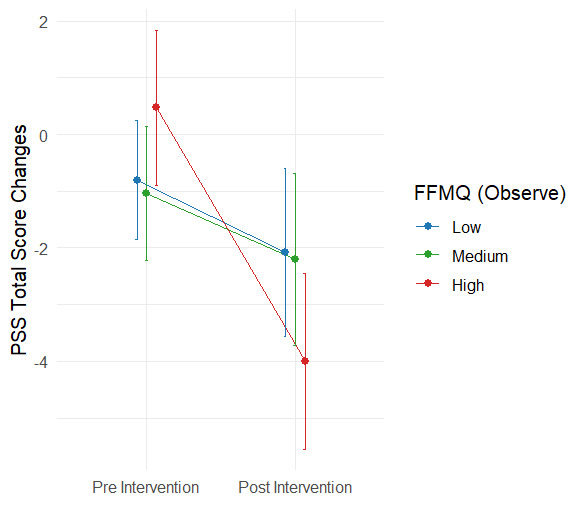
Changes in perceived stress using the 10-item Perceived Stress Scale (PSS-10) by levels of the 15-item Five Facet Mindfulness Questionnaire (FFMQ-15) observing facet. Low, medium, and high FFMQ—observing groups are defined using baseline tertiles. Error bars indicate 95% CI.

Additionally, the observing facet of mindfulness (FFMQ-15) also showed a significant moderation effect (B=−0.86, SE 0.41; *P*=.04). Health care professionals with higher baseline levels of mindful observation exhibited a more favorable response to the intervention. Further details of these analyses are presented in Table 2. Figure 3 illustrates the reductions in perceived stress during the preintervention and postintervention periods across 3 groups defined by baseline levels of the observing facet (low, medium, and high).

The results of the multivariate analysis are presented in Multimedia Appendix 1 and confirm the findings obtained in the univariate models. The moderating effects of baseline depressive symptoms (PHQ-9), nursing occupation, and the observing facet of mindfulness (FFMQ-15) on the effectiveness of the program remained statistically significant. In the multivariate model, these variables continued to play a significant moderating role in the reduction of perceived stress, whereas the remaining sociodemographic and occupational factors did not reach statistical significance.

In addition, the role of program adherence and the temporal sequence of the stepped-wedge design was explored. In the adjusted mixed-effects models, completing the program was not significantly associated with changes in perceived stress (B=−0.65, SE ##; *P*=.33), and no significant interaction was observed between the experimental condition and program completion (B=−0.99, SE 0.78; *P*=.20). Similarly, the temporal sequence of intervention implementation was not significantly associated with changes in perceived stress (B=−0.45, SE ##; *P*=.25) and did not moderate the intervention effect (B=0.61, SE ##; *P*=.19).

## Discussion

### Principal Findings

The present study explored individual, occupational, and psychological characteristics that may be associated with differential responses to the digital MINDxYOU program in reducing perceived stress among health care professionals. The findings suggest that baseline depressive symptoms, nursing occupation, and the observing facet of mindfulness may be potential moderators of intervention response. Although these results should be interpreted cautiously, they may provide preliminary insights into the characteristics of participants who could benefit most from this type of digital intervention. In this sense, the findings may contribute to ongoing efforts to develop more personalized and context-sensitive digital mental health programs for health care professionals.

First, the most robust finding of this study was the moderating role of baseline depressive symptoms. Participants with higher levels of depressive symptomatology at baseline benefited more from the intervention, showing larger reductions in perceived stress. This aligns with previous findings from an internet-delivered mindfulness-based cognitive therapy study [40], in which more severe baseline depression predicted greater posttreatment improvement in anxiety among cancer survivors. Although further research is needed, this suggests that individuals with greater initial distress may benefit more from online mindfulness-based interventions. This finding is consistent with the literature describing a bidirectional relationship between stress and depression, in which chronic stress acts as both a precipitating and a maintaining factor of depression, while depressive symptoms increase threat perception and reduce coping resources [41]. From this perspective, it is plausible that an intervention based on acceptance and commitment principles and on mindfulness, both of which have demonstrated efficacy in reducing depressive symptoms, may be particularly beneficial in this subgroup [42,43]. These interventions promote acceptance of internal experiences, psychological flexibility, and self-compassion, processes that counteract the cognitive mechanisms underlying depression, such as rumination and experiential avoidance [44]. Thus, participants with more severe depressive symptoms may have benefited more from acquiring adaptive coping and self-compassion strategies targeting processes common to both stress and depression. These findings suggest the potential usefulness of MINDxYOU for health care professionals with greater emotional vulnerability.

Second, professional occupation showed a significant moderating effect. The results indicated that the magnitude of change in perceived stress differed across professional groups, with nursing staff showing greater reductions after the intervention than physicians and other health care professionals. These findings should be interpreted cautiously, as the present analyses do not establish the mechanisms underlying these differences. Nevertheless, professional roles in health care may involve distinct emotional, organizational, and caregiving demands, which could influence how participants engage with and respond to stress-reduction interventions [2,6]. These findings highlight the importance of adapting the content and support structure of digital interventions according to the specific emotional demands associated with different health care roles.

Finally, the observing facet of mindfulness appeared to moderate the intervention effect, suggesting that observing may be associated with differences in response to the program. Previous evidence has shown that mindfulness facets may have differential associations with stress and anxiety responses: whereas acceptance-related facets were associated with lower subjective and cortisol stress reactivity, monitoring-related facets such as observing were associated with higher cortisol reactivity and anxious arousal [45,46]. This result may also complement previous mediation analyses of the MINDxYOU trial [12] that identified resilience and the describing and nonreactivity facets of mindfulness as potential mechanisms of change. Existing evidence suggests that mindfulness-related processes may operate differently depending on the clinical outcome, context, and level of practice, with some studies identifying emotional regulation processes as potential mechanisms linking mindfulness to anxiety and depression, while others show that increases in mindfulness facets may mediate the association between formal practice and improvements in stress, symptoms, and well-being [47,48]. However, evidence regarding the moderating role of the observing facet remains limited, and the present findings should therefore be considered preliminary. Future studies are needed to clarify whether baseline mindful observation contributes to differential responses in digital mindfulness-based interventions.

The digital format of the intervention may facilitate the implementation of personalization strategies in real-world health care settings. These findings are consistent with the idea that mindfulness-based and third-wave digital interventions may not be uniformly effective across all users and that their impact may vary according to psychological and contextual factors. From an applied perspective, this knowledge may inform the development of more personalized digital mental health programs, in which the level of guidance, support, or sequencing of content could be adapted to the user’s baseline profile [8]. Future studies should further investigate dynamic personalization mechanisms, integrating digital indicators of progress and hybrid clinical support to maximize the effectiveness of such interventions and enhance their real-world applicability in high-demand health care environments.

### Limitations

This study has several limitations. Although the stepped-wedge design and the use of mixed-effects models allow partial control of time-related effects, the influence of unmeasured variables cannot be ruled out. In addition, the sample consisted predominantly of women health care professionals working in the public sector, which may limit the generalizability of the findings to other professional or health care contexts. Furthermore, the use of self-report measures may introduce potential biases, such as social desirability or recall bias. Future studies should incorporate physiological indicators and objective measures of program use to provide a more comprehensive assessment of intervention effects. Additionally, the relatively large number of moderators that were examined increases the possibility of a type I error. Therefore, the findings should be interpreted cautiously and considered exploratory and hypothesis-generating until replicated in future confirmatory studies specifically designed to evaluate moderation effects. Finally, interactions between moderators and patterns of digital adherence were not examined. These aspects could be explored in future research using adaptive and personalized intervention models, which may help optimize the personalization and effectiveness of digital mental health interventions for health care professionals.

### Conclusions

The present study provides preliminary evidence that baseline psychological and occupational characteristics may influence responses to the MINDxYOU intervention among health care professionals. Higher depressive symptom scores, nursing occupation, and greater mindful observation were associated with larger reductions in perceived stress. These exploratory findings support the importance of considering individual characteristics when developing personalized digital mental health interventions.

## Supplementary material

10.2196/98624 Multimedia Appendix 1 Multivariate regression analyses examining the moderating effects of baseline depressive symptoms, nursing occupation, and the observing facet of mindfulness on intervention outcomes.

10.2196/98624 Checklist 1 CONSORT-eHEALTH checklist (V 1.6.1).

## Abbreviations

CD-RISC: Connor-Davidson Resilience Scale
FFMQ-15: 15-item Five Facet Mindfulness Questionnaire
PHQ-9: 9-item Patient Health Questionnaire
PSS-10: 10-item Perceived Stress Scale
