# Supplementary material for: Web-Based Mindfulness Intervention for Reducing Perceived Stress in Health Care Professionals: Secondary Analysis of Moderators in a Stepped-Wedge Cluster Randomized Trial
Source: JMIR Nurs. 2026 Aug 14;9:e98624. doi: 10.2196/98624 (PMC13476002; doi:10.2196/98624)
Supplement: Supplementary file 2 [file nursing-v9-e98624-s001.docx]

Multivariate Analysis

| **Moderator** | **B (estimate)** | **SE** | **df** | **t** | **p** |
| --- | --- | --- | --- | --- | --- |
| Condition | 7.61 | 3.19 | 745 | 2.39 | .017 |
| Sex | 1.26 | 0.80 | 511 | 1.58 | .12 |
| Age | 0.02 | 0.03 | 458 | 0.84 | .40 |
| Employment type (temporary) | 0.52 | 0.83 | 505 | 0.63 | .53 |
| Management position | -0.51 | 0.80 | 487 | -0.64 | .52 |
| Workplace setting (Primary care Center) | -1.19 | 0.83 | 508 | -1.43 | .15 |
| Workplace setting (Nursing Home) | 0.17 | 1.06 | 483 | 0.16 | .87 |
| Workplace setting (Other) | -0.89 | 0.75 | 523 | -1.19 | .24 |
| Occupation - Physician | 0.02 | 0.71 | 486 | 0.02 | .98 |
| Occupation-Other health care professionals | 0.36 | 0.84 | 519 | 0.43 | .67 |
| Baseline depression (PHQ-9) | -0.28 | 0.09 | 776 | -3.05 | .002 |
| Resilience (CD-RISC) | -0.05 | 0.06 | 745 | -0.84 | .40 |
| Mindfulness-Observing (FFMQ-15) | -1.13 | 0.44 | 768 | -2.59 | .010 |

Note. B= unstandardized regression coefficient; SE= standard error; df= degrees of freedom. All moderators were entered simultaneously in the multivariable mixed-effects model.
